# Hospitalization of very old critically ill patients in medical intermediate care units in France: a nationwide population-based study

**DOI:** 10.1186/s13613-025-01485-5

**Published:** 2025-05-27

**Authors:** Adrien Migeon, Arthur Kassa-Sombo, Emeline Laurent, Lucile Godillon, Leslie Grammatico-Guillon, Antoine Guillon

**Affiliations:** 1 Department of Geriatrics, Tours University Hospital, Tours, France; 2 Epidemiology Unit EpiDcliC, Clinical Data Center, Service of Public Health, Tours University Hospital, Tours, France; 3 Research Center for Respiratory Diseases (CEPR), INSERM U1100, University of Tours, 2 Bd Tonnellé, 37044 Tours Cedex 9, France; 4 Research unit EA1075 (Education, Ethics and Health), University of Tours, Tours, France; 5 Research unit MAVIVH, INSERM U1259, Medical School, University of Tours, Tours, France; 6 Intensive Care Unit, Tours University Hospital, University of Tours, Tours, France

**Keywords:** Critical care, Intensive care unit, Intermediate care units, Old patients, Long-term mortality

## Abstract

**Background:**

As the trajectory of very old critically-ill patients becomes an increasingly significant global challenge, these patients are often referred to intermediate care units. Intermediate care units provide a level of care that is less intensive than the intensive care unit (ICU) but more advanced than standard hospital wards. We aimed to assess the nationwide utilization of intermediate care units for critically ill patients aged 80 years or older (≥ 80 y.o.) and to examine their characteristics and long-term mortality outcomes.

**Methods:**

From the overall adult population (aged 18 years and older) hospitalized in France (French Hospital Discharge Database) from January 1, 2014, to December 31, 2022, patients ≥ 80 y.o. were included. We examined trends in the utilization of medical intermediate care units for critically ill patients ≥ 80 y.o and reported patient characteristics, including the Charlson comorbidity index and Hospital Frailty Risk Score. Readmission rates (hospital or rehabilitation unit) and mortality rates were calculated during a one-year follow-up period after the end of hospital stay.

**Results:**

The proportion of patients ≥ 80 y.o. in intermediate care units was 31% whereas it was 17% in ICU. Patients with greater comorbidities and severity were more frequently hospitalized in polyvalent intermediate care units (10% of them receiving acute organ support) compared to specialized intermediate care units. Admission to intermediate care units was associated with a 14% mortality rate during the stay, 28% at one year. Additionally, 58% of intermediate care units patients were rehospitalized within the year following discharge (6% in critical care units).

**Conclusions:**

One-third of the patients hospitalized in the intermediate care units in France are aged 80 years or older.

**Supplementary Information:**

The online version contains supplementary material available at 10.1186/s13613-025-01485-5.

## Introduction

Life expectancy has risen since industrialization, and 459 million octogenarians are expected in 2050 worldwide as compared with the current 155 million [1, 2]. In France, it is estimated that three new old people are added every five minutes, and as a result, the proportion of very old patients in intensive care units (ICU) has doubled over the past decade [3, 4]. However, the 1-year mortality for patients aged 80 years or older in ICU is close to 40%, and only a quarter of the survivors would recover their former autonomy [5]. Furthermore, very old patients represent a heterogeneous group, and their outcomes following a stay in critical care vary depending on multiple factors [6, 7]. A deterioration can be foreseeable according to the clinical severity, but also according to geriatric frailties [6–8]. Therefore, opting for a personalized decision to limit life-sustaining treatment, potentially restricting ICU admission, may be considered appropriate when no favorable outcome is anticipated [9]. In this context, intermediate care units (IMCU) offer the option of stepping up to ICU care or stepping down to general hospitalisation and represent a potential alternative. An international working group proposed a definition that distinguishes ICUs, which focus primarily on critically-ill patients, and IMCUs, which are intended for patients with serious but not critical conditions [10]. IMCUs have a level of nursing staff (and costs) lower than ICU although higher than in the general wards. In France, various types of IMCUs have been implemented since 2002, including organ-specific units such as cardiac, stroke, and hematological units, as well as multi-purpose units [11]. In a multicentre European cohort study, it was observed that the presence of an IMCU in the hospital was associated with significantly reduced adjusted hospital mortality for adults admitted to the ICU [12]. However, this effect was relevant only for the patients requiring full intensive treatment. Evidence supporting the use of IMCUs for critically-ill very old patients is limited. An ancillary analysis of the randomized clinical trial ICE-CUB2 suggested that admission to a medical ward was associated with worse 6-month survival in older critically-ill patients compared to admission to an IMCU, with no difference of survival between ICU and IMCU admissions [13]. The current policies for admitting critically-ill very old patients to medical wards, IMCUs, or ICUs are unclear. As a first step, there is a pressing need for a careful assessment of the use of intermediate care in this frail population. This study aimed to describe the characteristics and long-term mortality of patients aged of 80 years or older hospitalized in IMCU on a national scale.

## Methods

### Data source

A retrospective cohort was built using data from the French Hospital Discharge Database (HDD). All public and private French hospitals must provide a mandatory summary of each hospital stay, using diagnosis codes based on the International Classification of Diseases, tenth revision (ICD-10) and clinical procedure codes based on the French Current Procedural Terminology (CPT) (“*Classification Commune Des Actes Médicaux: CCAM*”). Each patient is assigned a unique identification number, enabling the reconstruction of their care pathway over time and facilitating epidemiological studies [4, 14–16].

### Study population

From the overall adult population (aged 18 years and older) hospitalized in France, we included all patients aged 80 years and older. We chose 80 years as a cutoff to define a very old patient for reasons previously detailed [17]. These patients were categorized based on their healthcare unit: polyvalent IMCU (P-IMCU), cardiac IMCU (C-IMCU), neurologic IMCU (N-IMCU), or ICU, as specified by the specific unit authorization (Supplementary Table S1). Patients admitted to both ICU and IMCU during the same hospital stay were classified in the ICU group. All other conventional medical units were aggregated into a single category. Patients in surgical critical care units, burn units, and those with organ transplants were not included in the study. Two study periods were defined. First, we analyzed a broader timeframe, including patients hospitalized from January 1, 2014, to December 31, 2022, to describe trends in IMCU admissions. Next, we focused on a representative two-year period, including patients hospitalized from January 1, 2017, to December 31, 2018, for a detailed analysis of their characteristics and one-year outcomes (hospital readmission and death).

### Variables of interest

The following variables were extracted for each inpatient stay: age, sex, site of admission and transfer(s), hospital death. Charlson comorbidity index (CCI) was calculated based on ICD-10 diagnoses [18–20]. The risk of frailty was estimated using the validated Hospital Frailty Risk Score (HFRS) for older people [14, 21]. The reason for admission was determined using the primary ICD-10 diagnosis recorded in IMCU or ICU and was subsequently classified by organ (Supplementary Table S2). Sepsis was defined when a sepsis ICD-10 code was reported as a primary or associated diagnosis. Since sepsis is defined syndromically across various medical conditions, we evaluated its incidence independently of organ-specific diseases. Specific care supports were identified using French CPT codes (Supplementary Table S3). SAPSII (Simplified Acute Physiology Score II) was extracted from the hospital resume. Data on the length of stay, overall and in IMCU or ICU, and the vital status at hospital discharge were also collected. Readmission rates to the hospital or rehabilitation unit were calculated for patients during a one-year follow-up period after the end of hospital stay. Rehospitalization was defined as a new hospital stay including a least one night and that was distant from at least 24 h from the initial stay. We furthermore studied the 1-year mortality rates, both for the IMCU and ICU groups, according to three starting points: (*i*) IMCU/ICU admission; (*ii*) IMCU/ICU discharge and (*iii*) end of hospital stay.

### Statistical analysis

Descriptive statistics were used (medians and frequencies) to summarize the data from the entire French population studied. Kaplan–Meier curves were performed to describe the 1-year survival. A sub-group analysis, by Cox regression models, was performed to assess risks of death after ICU and IMCUs admission according to age, sex, frailty risk status (assessed by HFRS), SAPSII and type of diagnosis. Data extraction and analyses were performed using SAS Enterprise Guide 8.3 ^®^ (SAS^®^ Institute, Cary, NC) and R 4.2.2, versions available on the national ATIH platform (“Agence Technique de l’Information sur l’Hospitalisation”) at the time of the study. Adherence to the STROBE guidelines for observational studies was maintained throughout the analysis [22].

### Ethics

Access to linked de-identified data in the HDD was performed in accordance with the French Reference Methodology procedure MR-005 for retrospective studies using HDD data, declaration signed by the teaching hospital of Tours (*MR005 number I4116221019*), as regulated by the French Data Protection Board *(Commission Nationale de l’Informatique et des Libertés, CNIL*). According to French data regulations, consent or information of each patient included was not required to use the French HDD de-identified data.

## Results

**Fig. 1:**
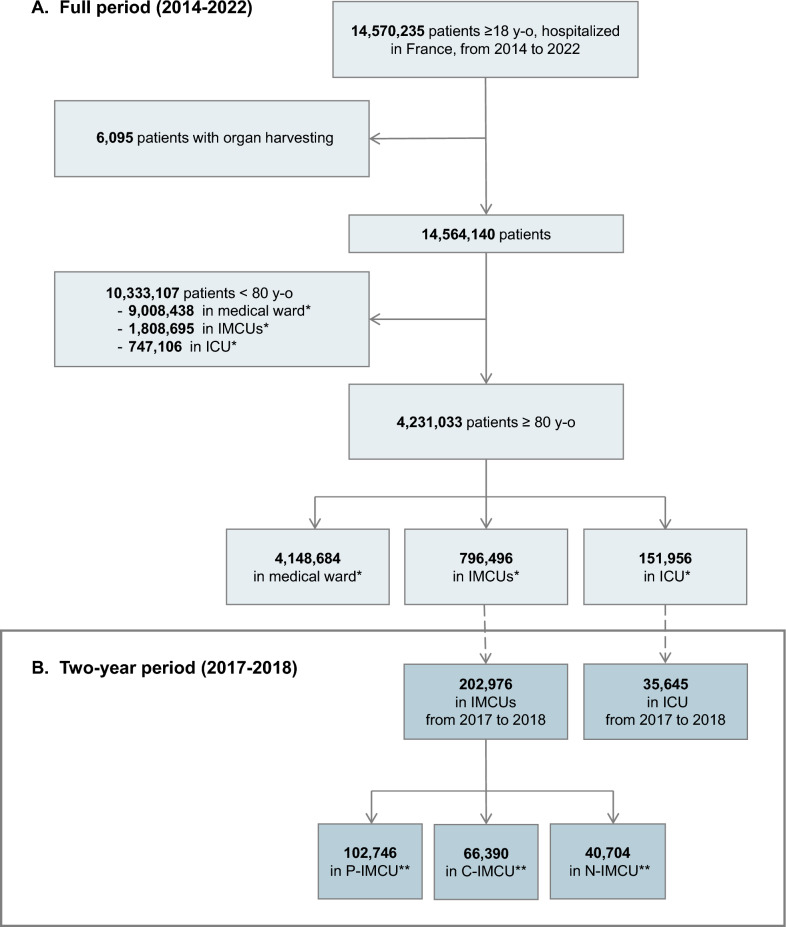
Flow chart of the population selection. **A** January 1, 2014, to December 31, 2022, France. **B** January 1, 2017, to December 31, 2018, France. Intensive care unit (ICU), Intermediate Care Unit (IMCU), cardiac IMCU (C-IMCU), neurologic IMCU (N-IMCU), or polyvalent IMCU (P-IMCU). * A patient could be admitted to medical ward and critical care during the same hospital stay. ** 6,864 patients (3.4%) have been admitted in several IMCUs types

**Fig. 2:**
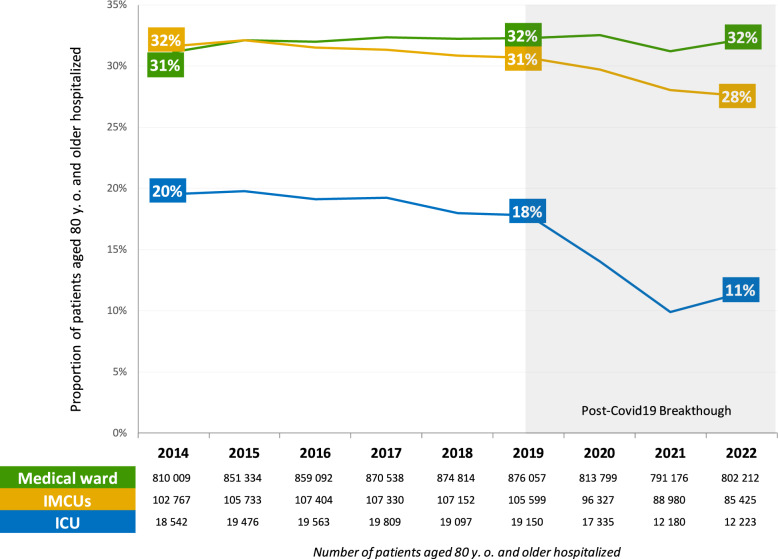
Proportion of very old patients (≥ 80 y.o.) hospitalized in the critical care units in France, from January 1, 2014, to December 31, 2022. The numbers in the table represent the absolute number of patients per year. Intensive care unit (ICU), Intermediate Care Unit (IMCU)

During the 2014–2022 period, a total of 948,452 individuals aged 80 and older were admitted to critical care: 796,496 in IMCU and 151,956 in ICU (Fig. 1). In IMCU, 31% of the patients were aged 80 years or older (respectively 30% in polyvalent units, 34% in cardiac units and 29% in neurologic units); in ICU, 17% of the patients were aged 80 years or older. In other medical wards, this proportion was 32%. The trends showed that while there was a drop in 2020–2021 of the proportion of patients ≥ 80 y.o. hospitalized in ICU, this drop was not observed in IMCU. The post-COVID-19 period did not return to what was observed before (Fig. 2).

**Fig. 3:**
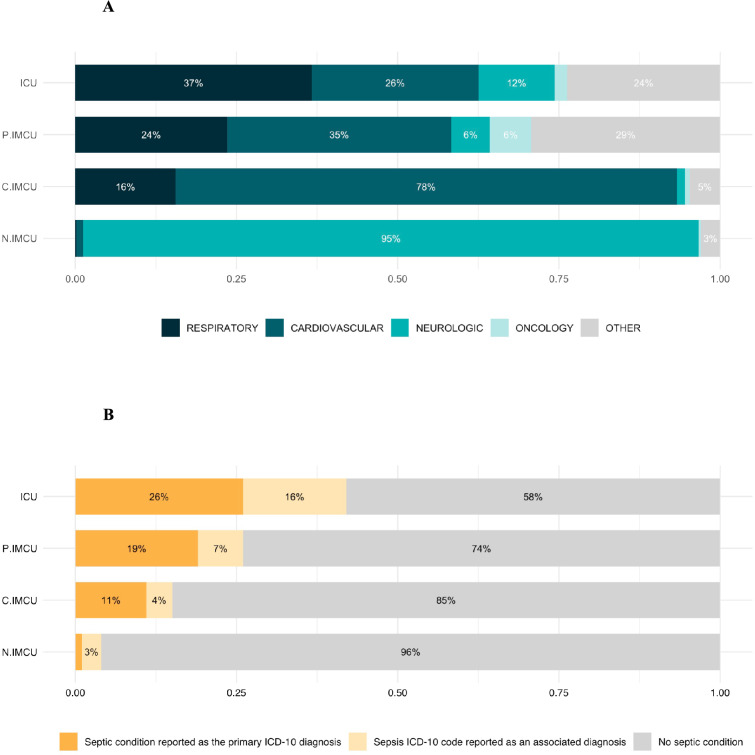
Admission diagnosis in IMCUs in France. **A** Primary diagnosis registered, **B** Sepsis as primary or secondary diagnosis. Intensive care unit (ICU), Intermediate Care Unit (IMCU), cardiac IMCU (C-IMCU), neurologic IMCU (N-IMCU), or polyvalent IMCU (P-IMCU)

During the two-year period (2017–18), a total of 238,621 individuals ≥ 80 y.o. were admitted to critical care: 202,976 in IMCU (85%) and 35,645 in ICU (15%) (Fig. 1B). Among patients ≥ 80 y.o., the primary diagnosis registered in IMCUs showed a balanced rates of primary diagnoses in polyvalent IMCU and a clear predominance of cardiovascular and neurologic diseases respectively in cardiac and neurologic IMCUs (Fig. 3A). Respiratory diseases were the main primary diagnoses for ICU (37%) and represented 24% of primary diagnoses in polyvalent IMCU. Sepsis affected 42% of ICU patients and 26% of patients in polyvalent IMCU (Fig. 3B).

The baseline characteristics of patients ≥ 80 y.o. are presented in Table 1. The proportions of frail or comorbid patients in polyvalent IMCU (25% of patients with CCI ≥ 3, 11% with HFRS ≥ 15) were similar to ICU and higher than in cardiac and neurologic IMCUs. Additionally, 27% of polyvalent IMCU patients had a SAPSII ≥ 40, which was less than in ICU (73%), but more than in other IMCUs (< 3%). More than 10% of polyvalent IMCU patients received acute organ supports as compared to less than 4% in other IMCUs. A large majority of IMCU patients were initially hospitalized via the emergency room, while direct admissions from home were less frequent (Table 1).

**Table 1:** Characteristics of patients aged 80 years or older hospitalized in critical care units in France

	ICU	All IMCUs	Polyvalent IMCU	Cardiac IMCU	Neurologic IMCU
	N = 35,645	N = 202,976	N = 102,746	N = 66,390	N = 40,704
Men (n, %)	18,840 (53%)	89,324 (44%)	47,064 (46%)	29,214 (44%)	16,277 (40%)
Age class (n, %)					
80–84 y.o	19,259 (54%)	82,135 (40%)	40,648 (40%)	26,754 (40%)	17,954 (44%)
85–89 y.o	12,390 (35%)	75,117 (37%)	37,758 (37%)	24,925 (38%)	14,849 (36%)
≥ 90 y.o	3,996 (11%)	45,724 (23%)	24,340 (24%)	14,711 (22%)	7,901 (19%)
Charlson Comorbidity Index (n, %)					
Low 0	17,351 (49%)	113,804 (56%)	51,684 (50%)	37,291 (56%)	28,706 (71%)
Middle [1, 2]	9,220 (26%)	47,166 (23%)	25,796 (25%)	15,321 (23%)	7,623 (19%)
High ≥ 3	9,074 (25%)	42,006 (21%)	25,266 (25%)	13,778 (21%)	4,375 (11%)
Hospital Frailty Risk Score (n, %)					
Low < 5	22,718 (64%)	139,863 (69%)	66,400 (65%)	47,892 (72%)	30,558 (75%)
Middle [5–15[	9,241 (26%)	44,520 (22%)	25,047 (24%)	13,638 (21%)	7,264 (18%)
High ≥ 15	3,686 (10%)	18,593 (9%)	11,299 (11%)	4,860 (7%)	2,882 (7%)
Pre-critical care unit location (n, %)					
Home	5,858 (16%)	38,533 (19%)	15,927 (16%)	174,40 (26%)	5,467 (13%)
Emergency department	22,497 (63%)	132,224 (65%)	65,755 (64%)	38,701 (58%)	31,578 (78%)
Medical ward	7,290 (21%)	32,219 (16%)	21,064 (21%)	10,249 (15%)	3,659 (9%)
SAPSII class (n, %)					
< 40	9,081 (27%)	174,919 (86%)	75,062 (74%)	64,561 (98%)	40,389 (99%)
[40–50[	7,547 (22%)	18,015 (9%)	17,226 (17%)	1,169 (2%)	185 (≤ 1%)
≥ 50	17,502 (51%)	10,042 (5%)	9,692 (10%)	455 (≤ 1%)	107 (≤ 1%)
Specific care supports (n, %)					
Vasopressor	15,366 (43%)	3,557 (2%)	3,565 (4%)	702 (≤ 1%)	37 (≤ 1%)
Median days [Q1-Q3]	3 [2–5]	2 [1–3]	2 [1–4]	2 [1–4]	2 [1–3]
Non-invasive ventilation	14,921 (42%)	12,972 (6%)	11,643 (11%)	2,172 (3%)	89 (≤ 1%)
Median days [Q1-Q3]	3 [2–6]	2 [1–5]	2 [1–5]	1 [1, 2]	1 [1–3]
Invasive ventilation	15,444 (43%)	1,384 (≤ 1%)	1,785 (2%)	226 (≤ 1%)	23 (≤ 1%)
Median days [Q1-Q3]	3 [2–8]	1 [1–4]	2 [1–6]	2 [1–6]	2 [1–6]
Length of stay in days					
In the unit (median [Q1-Q3])	4 [2–8]	3 [2–5]	3 [2–6]	3 [1–5]	3 [2–4]
In hospital (median [Q1-Q3])	13 [5–22]	9 [5–15]	10 [5–16]	8 [4–13]	9 [5–15]
Death during the stay (n, %)	15,278 (43%)	29,231 (14%)	18,558 (18%)	7,593 (11%)	4,496 (11%)

The 2-year study period was from January 1, 2017, to December 31, 2018

*ICU* Intensive Care Unit, *IMCUs* Intermediate Care Units, *P-IMCU* Polyvalent Intermediate Care Units, *C-IMCU* Cardiac Intermediate Care Units, *N-IMCU* Neurologic Intermediate Care Units

**Fig. 4:**
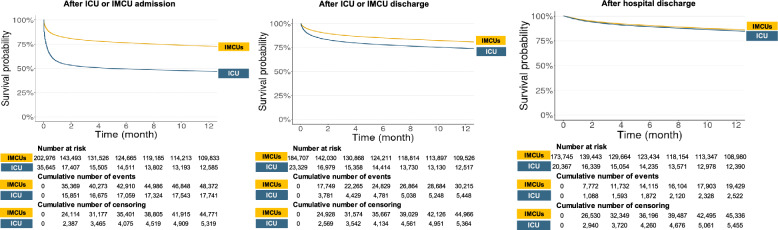
Kaplan–Meier curves showing the cumulative probabilities of survival, up to 12 months: after ICU or IMCU admission, after ICU or IMCU discharge, after hospital discharge. Intensive care unit (ICU), Intermediate Care Unit (IMCU)

Hospital death during the first stay was 14% for IMCU patients, ranging from 11% in cardiac and neurologic IMCUs to 18% in polyvalent IMCU, compared to 43% for ICU patients (Table 1, supplementary Table S4). The likelihood of death was adjusted for patient's condition at the time of admission, the presence of comorbidities and the risk of frailty (Table 2). The overall one-year mortality after hospital admission was 28% for patients ≥ 80 y.o. hospitalized in IMCU, as compared to 54% for those hospitalized in ICU (Fig. 4A, supplementary Table S4). The survival curves were biphasic, suggesting an important mortality rate in the first weeks or months following discharge, then stabilized. The gap between IMCU and ICU in one-year mortality tended to narrow when considering patients after discharge from critical units (Fig. 4B). Ultimately, there was no clinically relevant difference in one-year mortality among patients discharged alive, regardless of whether they were initially admitted to the ICU or IMCU (Fig. 4C). Details for one-year mortality according to the category of IMCU are provided in Supplementary Fig. 1.

**Table 2:** Hazard ratios for risk of death after ICU and IMCUs admission from adjusted Cox regression models

		ICU		P-IMCU		C-IMCU		N-IMCU	
		(N = 35,645)		(N = 102,746)		(N = 66,390)		(N = 40,704)	
Characteristics		HR	95% CI	HR	95% CI	HR	95% CI	HR	95% CI
Age	80–85 y.o	ref		ref		ref		ref	
	85–89 y.o	1.13	[1.10–1.16]	1.23	[1.20–1.26]	1.35	[1.31–1.39]	1.43	[1.37–1.50]
	≥ 90 y.o	1.39	[1.33–1.46]	1.63	[1.59–1.68]	1.89	[1.82–1.95]	2.16	[2.05–2.28]
Sex	Female	ref		ref		ref		ref	
	Male	1.17	[1.14–1.20]	1.36	[1.33–1.38]	1.44	[1.40–1.48]	1.36	[1.30–1.41]
Frailty	HFRS < 5	ref		ref		ref		ref	
	HFRS 5–15	1.12	[1.09–1.16]	1.28	[1.25–1.31]	1.42	[1.38–1.47]	1.44	[1.37–1.51]
	HFRS ‚â• 15	1.24	[1.18–1.29]	1.50	[1.45–1.54]	1.65	[1.57–1.73]	1.78	[1.67–1.91]
SAPSII	< 40	ref		ref		ref		ref	
	40–49	1.31	[1.26–1.37]	1.57	[1.53–1.61]	1.74	[1.57–1.93]	1.84	[1.39–2.44]
	≥ 50	3.02	[2.91–3.13]	3.54	[3.45–3.64]	3.81	[3.33–4.37]	6.27	[4.72–8.32]
Sepsis	No	ref		ref		ref		–	
	Yes	0.87	[0.84–0.90]	0.91	[0.89–0.94]	0.83	[0.79–0.87]	–	
Diagnostic	Respiratory	1.24	[1.19–1.29]	1.41	[1.38–1.45]	–		–	
	Cardiovascular	1.51	[1.45–1.57]	1.12	[1.09–1.14]]	–		–	
	Neurologic	1.54	[1.47–1.61]	1.17	[1.12–1.22]				

The 2-year study period was from January 1, 2017, to December 31, 2018. Cox models were performed by age, sex, risk of frailty, SAPSII, sepsis and type of diagnosis. Cardiovascular and neurologic diseases were not included in Cox regression models for C-IMCU and N-IMCU respectively. Sepsis was not considered for Cox regression model in N-IMCU due to its negligible occurrence

*ICU* Intensive Care Unit, *IMCUs* Intermediate Care Units, *P-IMCU* Polyvalent Intermediate Care Units, *C-IMCU* Cardiac Intermediate Care Units, *N-IMCU* Neurologic Intermediate Care Units

Results regarding outcomes and rehospitalization rates of patients ≥ 80 y.o. being hospitalized in critical care in France are presented in Table 3 and supplementary Fig. 2. Rates of re-admission in acute care settings at one year were similar in IMCU and ICU populations: 58% and 61%, respectively; with 6% and 7% of readmissions in critical care units, respectively (Table 3).

**Table 3:** One-year outcomes of patients aged 80 years or older after being hospitalized in critical care units in France

	ICU	All IMCUs	Polyvalent IMCU	Cardiac IMCU	Neurologic IMCU
	N = 20,367	N = 173,745	N = 84,188	N = 58,797	N = 36,208
Hospitalization in a rehabilitation unit (n, %)	9,345 (46%)	58,616 (34%)	28,270 (34%)	17,364 (30%)	15,141 (42%)
Re-admission in hospital (n, %)	12,474 (61%)	99,917 (58%)	51,094 (61%)	35,959 (61%)	16,134 (45%)
Number of re-admissions per patient (median, [Q1-Q3])	2 [0–3]	1 [0–3]	1 [0–3]	1 [0–3]	1 [0–3]
Of which, ICU or IMCU re-admission	1,458 (7%)	11,072 (6%)	6,422 (8%)	4,099 (7%)	1,033 (3%)
Of which, ICU or IMCU re-admission for the same organ failure	622 (3%)	5,447 (3%)	2,791 (3%	2,449 (4%)	478 (1%)

The 2-year study period was from January 1, 2017, to December 31, 2018

*ICU* Intensive Care Unit, *IMCUs* Intermediate Care Units, *P-IMCU* Polyvalent Intermediate Care Units, *C-IMCU* Cardiac Intermediate Care Units, *N-IMCU* Neurologic Intermediate Care Units

Moreover, as the ICU, polyvalent-ICU and cardiac ICU were we very close trend of rehospitalization within the first year after hospital discharge, the neurological ICU seems to be quite different with a very lower rate of hospital readmission (Supplementary Fig. 2). The first 3 months after discharge seems to be a very high-risk period for very old people discharged for medical reasons, in relation to the initial trend in the incidence of death. However, in contrast to the mortality incidence, the hospitalization cumulative incidence decreases from month 3 to the end of the first year, although it remained substantial (Supplementary Fig. 2).

## Discussion

IMCU have emerged as a crucial resource to critically-ill patients, providing high-quality treatment with continuous care. Because the population aging is an unprecedented challenge for healthcare systems, IMCUs are extensively used for very old and critically-ill patients. We demonstrated that nearly 30% of IMCU patients hospitalized in France are aged of 80 years and older (nearly 100,000 patients per year). Among these very old patients, 10% received acute organ supports such as vasopressor infusion or mechanical ventilation in polyvalent IMCU. Admission to IMCUs was associated with a mortality rate of 14% during the stay and a 1-year mortality rate of 28% for these old patients.

Interest in IMCUs has increased in both Europe and United States; however, comparisons are complicated due to differences in definitions [23]. In France, polyvalent IMCU are dedicated to patients who are at risk of developing one or more acute failures that may require temporary life-support methods, and transfer to the ICU. Our findings demonstrated important differences between very old patients hospitalized in polyvalent or specialized IMCUs. Patients hospitalized in polyvalent IMCUs were frailer, more comorbid, more critically-ill, and required more specific care supports compared to patients hospitalized in specialized IMCUs.

The appropriate management of the very old patient is an important and pressing challenge. Interestingly, our analysis revealed that the policies for admitting these patients were likely to be similar in medical wards and IMCU. On the contrary, admission policies were more restrictive in ICU for very old and critically-ill patients compared to IMCU. For instance, the sharp increase in mortality among older patients with COVID-19 [17] resulted in a significant reduction in ICU admissions for very old patients, while IMCU admissions remained poorly affected. The primary goal of the IMCU is to ensure long-term survival while achieving the highest possible quality of life. The recently published systematic review on the utility of IMCU highlighted that there is a knowledge gap regarding the long-term mortality after IMCU [23].

The concept of geriatric frailty appears to be one of the most important mechanisms to be understood for older people in ICUs [6]. The HFRS has the potential to be widely and automatically used in a time and cost-effective manner, without inter-user variability, and was used in this study to assess the risk of frailty [14, 24]. However, some limitations should be highlighted. First, it is important to note that the HFRS has been designed to assess the risk of frailty, but not the severity of the frailty itself [21]. In addition, the HFRS is based on electronically ICD-10 codes, which are associated with poor outcomes and organ system involvement in older patients [21]. Therefore, it may be limited by incomplete or misreported data. Furthermore, ICD-10 codes have historically been used for reimbursement and are not designed to reflect disease severity, polypharmacy, or environmental factors, which could contribute to reduced physiological capacity to respond to an acute situation, the most widely accepted definition of frailty, which was not included in the HFRS [25].

By studying the French population at a national scale, we observed that one-quarter of the very old patients hospitalized in IMCU died within one year after admission. Interestingly, when we analysed the one-year mortality of hospital survivors after discharge, we found that the mortality rate and hospital re-admission were identical for very old patients, regardless of whether they were initially hospitalized in the IMCU or ICU. These findings suggest that long-term prognosis in this age group may be primarily determined by underlying frailty and disease burden, rather than by the intensity of the acute illness. This perspective highlights the need for more accurate prognostic tools that extend beyond acute illness severity and instead focus on patients' baseline status. Developing such tools could lead to more personalized treatment plans and better allocation of resources, ultimately improving outcomes for very old patients facing complex health challenges [26, 27]. Moreover, our results suggest that IMCUs are a viable alternative to ICUs for very old critically ill patients, offering potential benefits in comfort and cost-effectiveness. They may provide a balanced approach to care, especially for patients who do not require intensive organ support.

This study should be read with an understanding of its methodological choices and limitations. (i) Patients can transit between the ICU and IMCU during the same hospitalization. To clarify the policy for admitting very old patients to the IMCU, we classified patients in the IMCU group only if their stay was not associated with an ICU stay. Thus, the overall number of very old patients hospitalized in IMCU is higher if IMCU hospitalization before/after ICU stay are considered. (ii) Surgical patients were not included in this study because the prognosis for planned perioperative situations is already well-documented and differs significantly from acute organ dysfunction in very old patients [28, 29]. (iii) The 2014–2022 period was used to provide the global trend of very old patient hospitalized in IMCU. The 2017–2018 period was selected for detailed analysis. We chose to exclude the COVID-19 pandemic period because it was not representative of usual conditions, and the post-pandemic period because alterations in care pathways had not returned to pre-COVID-19 conditions. Additionally, the 2014–2016 period was necessary to gain the most comprehensive insights into patient characteristics prior to their hospitalization in the intermediate care unit in 2017. (iv) It is unknown, using this methodology, whether the patients hospitalized in the IMCU had "do not resuscitate" orders and/or were denied ICU admission. Similarly, deaths in IMCU preceded by a decision to withhold or withdraw life sustaining treatment could not be identified. (v) The codes assigned to high-flow oxygen therapy were inconsistent throughout the study period. Due to this coding imprecision, we chose not to include this therapy in our analysis, despite its well-established importance in current clinical practice. (vi) The quality-of-life post-discharge could not be directly assessed. As a surrogate measure, we calculated the rate of readmission within the following year, assuming that unresolved or worsening condition is likely to be associated with poor quality of life [30, 31]. We found that an important proportion of IMCU patients (58%) were rehospitalized during the year following discharge and 6% were re-admitted in critical care units.

In conclusion, we found that nearly one-third of patients directly admitted to the IMCU in France were very old (≥ 80 y.o.). We believe that this epidemiological information is crucial for further demonstrating that demographic aging has a growing impact in intensive care, including IMCUs. This trend highlights the need for healthcare systems to adapt and optimize care strategies tailored specifically for an aging population, ensuring that resources are allocated efficiently to meet their unique medical needs.

## Supplementary Information

Additional file 1.: Figure 1. Kaplan-Meier curves showing the cumulative probabilities of survival, up to 12 months: after intensive care unit (ICU), cardiac intermediate care unit (C-IMCU), neurologic IMCU (N-IMCU), or polyvalent IMCU (P-IMCU).

Additional file 2.: Figure 2. Kaplan-Meier curves showing the cumulative probabilities of re-hospitalization, up to 12 months after hospital discharge. Polyvalent Intermediate Care Unit (P-IMCU), Cardiac Intermediate Care Unit (C-IMCU), Neurologic Intermediate Care Unit (N-IMCU), Intensive care unit (ICU).

Additional file 3.

## Data Availability

Restrictions apply to the availability of these data and so are not publicly available. However, aggregated data are available from the authors upon reasonable request and with the permission of the institution, or via the national Hub ATIH (https://www.atih.sante.fr/).
