# Supplementary material for: Hospitalization of very old critically ill patients in medical intermediate care units in France: a nationwide population-based study
Source: Ann Intensive Care. 2025 May 27;15:73. doi: 10.1186/s13613-025-01485-5 (PMC12116954; doi:10.1186/s13613-025-01485-5)
Supplement: Supplementary file 1 — Additional file 1.: Figure 1. Kaplan-Meier curves showing the cumulative probabilities of survival, up to 12 months: after intensive care unit (ICU), cardiac intermediate care unit (C-IMCU), neurologic IMCU (N-IMCU), or polyvalent IMCU (P-IMCU). [file 13613_2025_1485_MOESM1_ESM.pptx]

## Slide 1
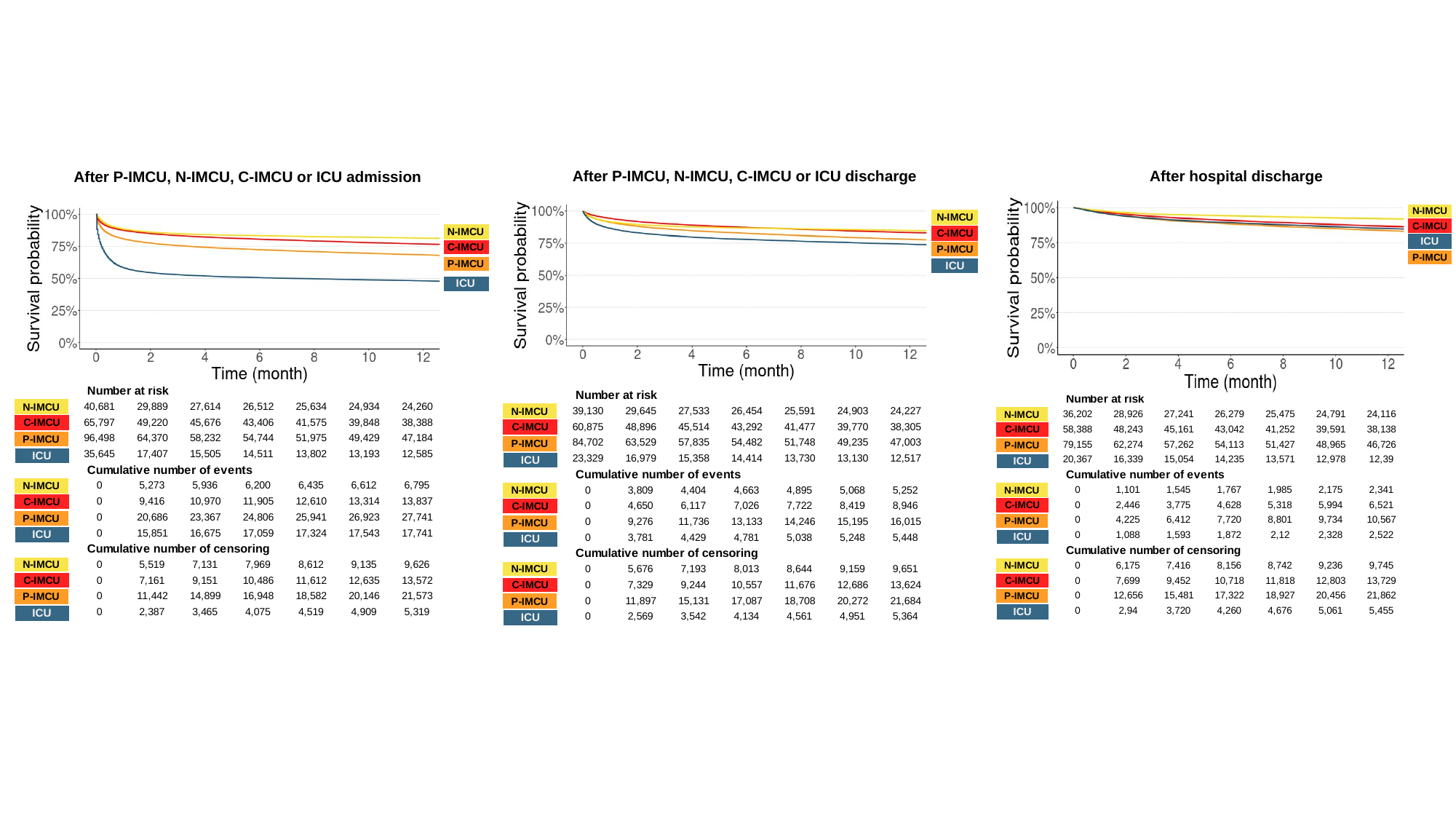

After hospital discharge
After P-IMCU, N-IMCU, C-IMCU or ICU discharge
After P-IMCU, N-IMCU, C-IMCU or ICU admission
