# Supplementary figures and images for: Hospitalization of very old critically ill patients in medical intermediate care units in France: a nationwide population-based study
Source: Ann Intensive Care. 2025 May 27;15:73. doi: 10.1186/s13613-025-01485-5 (PMC12116954; doi:10.1186/s13613-025-01485-5)

## Slide 1
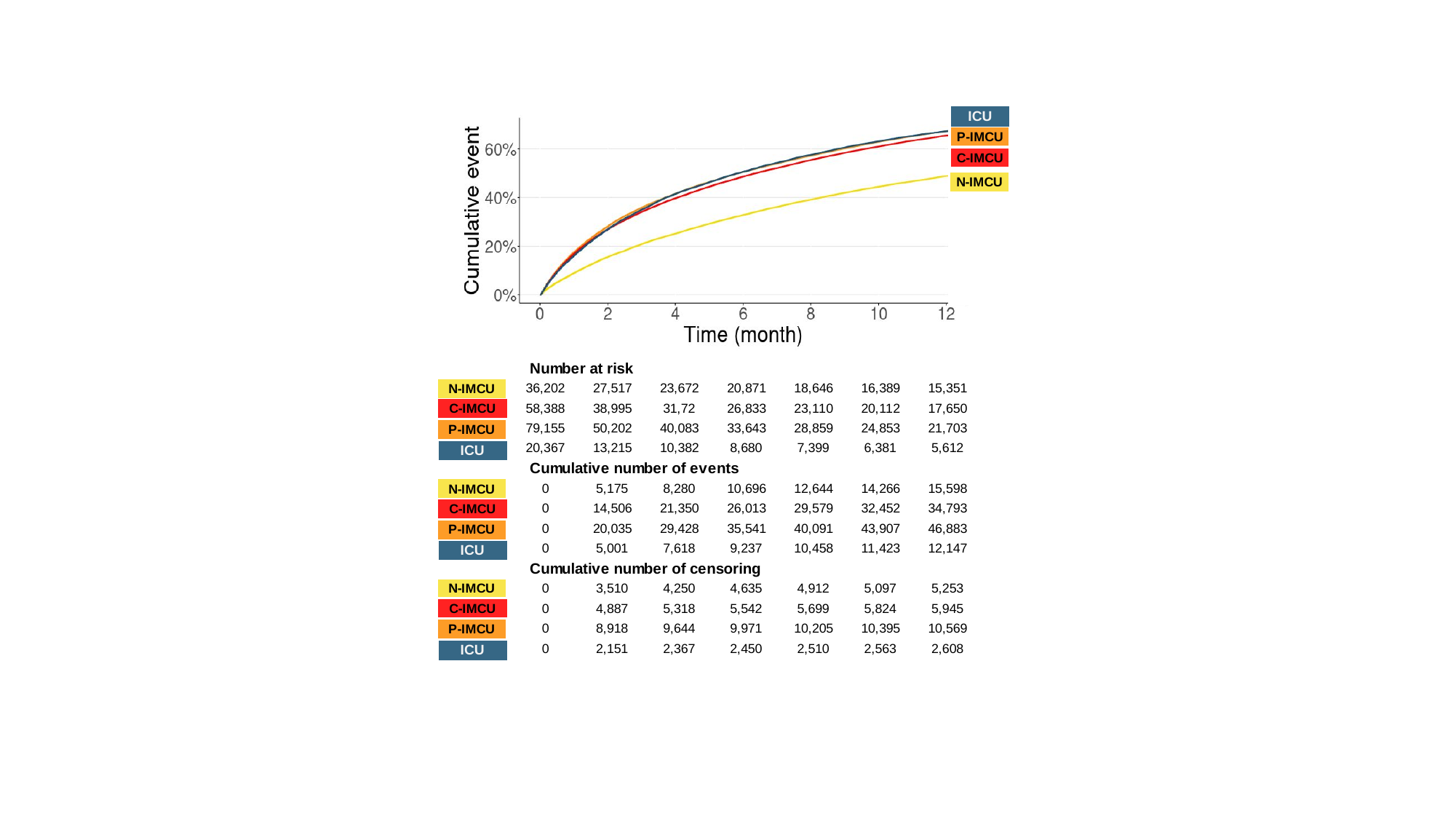

Supplement: Supplementary file 2 — Additional file 2.: Figure 2. Kaplan-Meier curves showing the cumulative probabilities of re-hospitalization, up to 12 months after hospital discharge. Polyvalent Intermediate Care Unit (P-IMCU), Cardiac Intermediate Care Unit (C-IMCU), Neurologic Intermediate Care Unit (N-IMCU), Intensive care unit (ICU). [file 13613_2025_1485_MOESM2_ESM.pptx]
