# Supplementary material for: Hospitalization of very old critically ill patients in medical intermediate care units in France: a nationwide population-based study
Source: Ann Intensive Care. 2025 May 27;15:73. doi: 10.1186/s13613-025-01485-5 (PMC12116954; doi:10.1186/s13613-025-01485-5)
Supplement: Supplementary file 3 — Additional file 3. [file 13613_2025_1485_MOESM3_ESM.docx]

**Supplementary materials: Tables**

**Table S1.** French healthcare unit specific authorization.

**Table S2.** Organ classification of ICD-10 primary diagnosis.

**Table S3.** French CPT codes of specifics ICU care supports.

**Table S4.** Mortality distribution, patients ≥ 80 y.o. 2017-2018.

**Table S1.** French healthcare unit specific authorization

| **Code of medical unit** | **Labels** |
| --- | --- |
| 01A | Adult intensive care, excluding severe burns ICU |
| 02A | Intensive cardiac care = CCU (Cardiac Care Unit) |
| 02B | Other intensive care (excluding Stroke Units, CCU, and Neonatology) |
| 03A | Adult intermediate care, excluding severe burns intermediate care unit |
| 18 | Intensive care in Stroke Unit |

*Intensive Care Unit (ICU)*

**Table S2.** Organ classification of ICD-10 primary diagnosis.

| **ICD-10 classification by organ** | **List of ICD-10 codes** |
| --- | --- |
| CARIDOVASCULAR | E86, E86, I00-I25, I300, I301, I30-I31, I328, I34-I37, I401-I409, I418-I42, I430, I431-I519, I70-I721, I723-I729, I74, I77, I790-I791, I792-I80, I821-I822, I828-I829, I95, I970-I971, I978-I979, L97, R00-R01, R03, R55, R570-R571, R578, R579, R58, T78, T811 |
| CARDIOVASCULAR septic | A021, A327-A329, A392-A395, A40, A41, A427, A440, A481-A484, A483, A75-A79, A90-A99, B332, B334, B377, B464, B49, B570, B572, I320-I321, I33, I38-I400, I410-I412, I52, I980-I981, J853, J853, R572 |
| RESPIRATORY | E840, J30-J31, J33, J341-J35, J38, J40-J439, J441-J47, J60-J70, J80, J81-J84, J92-J95, J960, J961-J99, R04-R091, R092, U04 |
| RESPIRATORY septic | A15-A16, A19, A310, A36-A38, A420, A430, A481, A691, A70, B012, B052, B250, B342, B371, B44, B450, B460, B510, B55, B573, B583, B59, I26-28, J00-J06, J09-J18, J20-J22, J32, J340, J36-J37, J39, J440, J850-J852, J86, J90-J91, R093-R094 |
| NEUROLOGY | E100, E140, E15, G08-G09, I60-I680, I682-I69, R20, R25-R29, R40-R49, R51-R52, R56, R90 |
| NEUROLOGY septic | A17, A321, A390, A80-A89, B003-B004, B010-B011, B020-B023, B050-B021, B261-B262, B375-B376, B451, B461, B500, B56, B571, B574, B582, B690, G00-G07, I681 |
| HEMATOLOGY and ONCOLOGY | C00-D97, R91-R92 |
| OTHERS | R96, R99, E841, I81, I820, I85, I982, K20-K31, K35-K38, K40-K46, K50-K52, K55, K56-K63, K66, K70-K76, K80, K85-K86, K90-K93, R10-R19, R74, N30, N33-N34, N390, N41, N45, N51, N61, N70-N77, D80-D89, E102, E112, E132, E142, E83-E85, E87, I722, I73, I78, I823, L50-L54, L93-L95, M05-M14, M30-M36, M359, M60-M61, M63, N00-N079, N081-N087, N089, R50, R79, B20-B24, B520, I78, N080, N088, T96-T97, E00-E059, E061-E07, E101, E111, E121, E131, E141, E20-E35, E40-E64, E70-E72, E74-E80, E88-E89, R63-R64, L10-L14, M15-M25, M40-M54, M62, M65-M79, M80-M99, R02, R21-R22, S00-T35, T79, T90-T95, T98, F00-F99, R78, T51-T57, X60-X94, X85-Y09, T36-T50, T80-T801, T803-T813, T815-T825, T828-T834, T837-T844, T848-T856, T858-T873, T875-T879, T881-T88, Y40-Y84 |
| OTHERS septic | A00-A020, A022-A09, A421, B007-B009, B15-B19, B251-B259, B263, B27, B462, B581, B65-B689, B691-B83, D73, K65, K67, K77, K81-K83, K87, A18, A20-A28, A30, A311-A319, A32, A398-A399, A428-A429, A438-A439, A448-A449, A488, A49, B005, B018-B019, B027-B029, B03, B04, B053-B059, B09, B268, B551-B559, B575, B588-B589, B95-B97, B508-B509, B518-B519, B528-B529, B53-B54, B99, N31-N32, N35-N389, N391-N399, N60-N609, N611-N64, N92-N93, R30-R39, A391, E060, E16, A422, A431, A441, A46, A480, B000-002, L00-L08, M00-M03, T802, T814, T826-T827, T835-T836, T845-T847, T857, T874, T880 |

*International Classification of Diseases (ICD-10)*

**Table S3:** French classification of procedures terminology (CPT) for specific ICU care supports.

| **CPT procedure** | **Codes** | **Labels** |
| --- | --- | --- |
| Vasopressor | EQLF001 | Continuous intravenous injection of dobutamine or dopamine at a rate of less than 8 micrograms per kilogram per minute [µg/kg/min], or dopexamine outside the neonatal period, per 24 hours |
|  | EQLF003 | Continuous intravenous injection of dobutamine or dopamine at a rate exceeding 8 micrograms per kilogram per minute [µg/kg/min], adrenaline or noradrenaline outside the neonatal period, per 24 hours |
| Non-Invasive mechanical ventilation CPAP | GLLD003 | Spontaneous ventilation by face mask, nasal cannula or nasopharyngeal tube, without inspiratory support, with positive expiratory pressure [VS-PEP] [Continuous positive airway pressure] [CPAP], per 24 hours |
| Non-Invasive mechanical ventilation BPAP | GLLD019 | Non-invasive barometric or volumetric ventilation by face mask for at least 2 hours cumulative over a 12-hour period, for acute respiratory failure |
|  | GLLD012 | Continuous mechanical ventilation with face mask for ventilatory support, per 24 hours |
| Invasive mechanical ventilation | GLLD015 | Intratracheal mechanical ventilation with positive expiratory pressure [PEEP] less than or equal to 6 and FiO2 less than or equal to 60%, per 24 hours |
|  | GLLD008 | Intratracheal mechanical ventilation with positive expiratory pressure [PEEP] greater than 6 and/or FiO2 greater than 60%, per 24 hours |
|  | GLLD004 | Intratracheal mechanical ventilation with positive expiratory pressure [PEEP] greater than 6 and/or FiO2 greater than 60%, with alternate prone positions every 24 hours |

**Table S4.** Mortality of patients aged 80 years or older hospitalized in critical care units in France in 2017-2018.

|  | **ICU** | **All IMCUs** |
| --- | --- | --- |
|  | N = 35,645 | N = 202,976 |
| In ICU or IMCUs | 12,362 (35%) | 18,507 (9%) |
| From ICU or IMCUs discharge to the hospital discharge | 2,916 (8%) | 10,724 (5%) |
| From hospital discharge to the first 6 months after admission* | 1,784 (5%) | 13,684 (8%) |
| From 6 month after admission to 12 months after admission** | 681 (2%) | 5,458 (3%) |
| Overall mortality** | 17,743 (54%) | 48,373 (28%) |
| *Intensive Care Unit (ICU); Intermediate Care Units (IMCUs)*  **Without missing data at 6 months N=2,234 in ICU; N=23,730 in All IMCUs*  ***Without missing data at 12 months N=2,607 in ICU; N=27,559 in All IMCUs* | | |
